# Predictive Modelling of Lung Function using Emphysematous Density Distribution

**DOI:** 10.1038/s41598-019-56351-9

**Published:** 2019-12-24

**Authors:** Kuo-Lung Lor, Cheng-Pei Liu, Yeun-Chung Chang, Chong-Jen Yu, Cheng-Yi Wang, Ming-Jui Chung, Fan-Ya Lin, Chung-Ming Chen

**Affiliations:** 1 Department of Biomedical Engineering, National Taiwan University, No. 1, Sec. 1, Jen - Ai Rd., Taipei City, 100 Taiwan (R.O.C.); 2 National Taiwan University Hospital, No. 7, Chung Shan S. Rd., Zhongzheng Dist., Taipei City, 10002 Taiwan (R.O.C.); 3 Cardinal Tien Hospital, No. 362, Zhongzheng Rd., Xindian Dist., New Taipei City, 23148 Taiwan (R.O.C.)

**Keywords:** Computational models, Chronic obstructive pulmonary disease

## Abstract

Target lung tissue selection remains a challenging task to perform for treating severe emphysema with lung volume reduction (LVR). In order to target the treatment candidate, the percentage of low attenuation volume (LAV%) representing the proportion of emphysema volume to whole lung volume is measured using computed tomography (CT) images. Although LAV% have shown to have a correlation with lung function in patients with chronic obstructive pulmonary disease (COPD), similar measurements of LAV% in whole lung or lobes may have large variations in lung function due to emphysema heterogeneity. The functional information of regional emphysema destruction is required for supporting the choice of optimal target. The purpose of this study is to develop an emphysema heterogeneity descriptor for the three-dimensional emphysematous bullae according to the size variations of emphysematous density (ED) and their spatial distribution. The second purpose is to derive a predictive model of airflow limitation based on the regional emphysema heterogeneity. Deriving the bullous representation and grouping them into four scales in the upper and lower lobes, a predictive model is computed using the linear model fitting to estimate the severity of lung function. A total of 99 subjects, 87 patients with mild to very severe COPD (Global Initiative for Chronic Obstructive Lung Disease (GOLD) stage I~IV) and 12 control participants with normal lung functions (forced expiratory volume in one second (FEV_1_)/forced vital capacity (FVC) > 0.7) were evaluated. The final model was trained with stratified cross-validation on randomly selected 75% of the dataset (n = 76) and tested on the remaining dataset (n = 23). The dispersed cases of LAV% inconsistent with their lung function outcome were evaluated, and the correlation study suggests that comparing to LAV of larger bullae, the widely spread smaller bullae with equivalent LAV has a larger impact on lung function. The testing dataset has the correlation of r = −0.76 (p < 0.01) between the whole lung LAV% and FEV_1_/FVC, whereas using two ED % of scales and location-dependent variables to predict the emphysema-associated FEV_1_/FVC, the results shows their correlation of 0.82 (p < 0.001) with clinical FEV_1_/FVC.

## Introduction

The two main components of COPD are chronic bronchitis and emphysema^1,2^. The consequence of the disturbances of gas exchange at the alveolar level caused by bronchitis and emphysema diminishes patients’ life quality when they frequently experience shortness of breath^3–6^. The diagnosis of COPD in patients with exposure to risk factors, such as smoking can be confirmed by spirometry. The pulmonary function test (PFT) of spirometry provides measurements of, among others, forced expiratory volume in one second (FEV_1_) and forced vital capacity (FVC). FEV_1_and FVC is the two main measurements for the diagnosis of COPD. While PFT is an effective gold standard for the diagnosis of COPD, this criterion cannot objectively reflect regional pulmonary destruction, but the overall clinical presentation of lung function. In contrast to the PFT, CT images illustrate the progression of emphysematous lesions with low lung density. By measuring the percentage of low attenuation volume (LAV%) using a fixed threshold of HU, this clinical gold standard, however, cannot reflect the heterogeneity of emphysema^7^. COPD patients with similar measurements of PFT, may have a discrepancy between their LAV% and vice versa. One of the new clinical demands of emphysema assessment is the initial evaluation of lung volume reduction (LVR), which requires accurate and objective characterization methods for the regional distribution of emphysema on quantitative CT.

LVR is the surgical resection of advanced emphysematous lung tissue while preserving the more functional regions to restore elastic recoil and radial traction on the terminal bronchioles^8^. The National Emphysema Treatment Trial (NETT) indicated that patients with non-upper lobe emphysema have a higher operative mortality rate than those with upper lobe predominant emphysema^9^. Other new techniques of endobronchial LVR (ELVR) which are less invasive, place valves and coils to reduce inspiratory airflow to the target lobe. The selection criteria of the target lobe are based on the evaluation of lobar LAV%, interlobar fissure completeness and emphysema heterogeneity^10,11^. Other pathophysiological assessment includes the identification of the contraindications such as large bullae for LVR. However, the current selection criteria are not based on the prediction of the treatment response to the lung function at the lobar level while the treatment is usually focused on a single lobe^12^. Several studies have shown that the emphysema of the lower lobe has more influence on lung function^13–15^. Some studies claimed that the overall lung function would be preserved with the absence of emphysema in lower lobe and centrilobular emphysema correlates better with the results of PFT^15–18^. More studies have suggested the estimation of at least 30% of emphysematous destruction before symptoms or lung function abnormalities become evident^19^. The extent of total or lobar LAV% can only reveal the proportions of destruction within the area, but such proportions may not relatively correspond to the equivalent loss of lung function. Studies have shown that the densitometric data do not predict the ventilation, such that the most emphysematous lobe does not necessarily have the least ventilation^11,12^. Hetzel *et al*. developed a new functional method to choose the target lobe for LVR based on the lobar vital capacity/lobar total capacity, but the method is limited to the lobar level evaluation and not all LVR techniques treat whole lobes. Developing an accurate and objective predictive model for the lung function outcome base on the regional distribution of intra-lobar emphysema on quantitative CT is therefore needed.

The overall aim of this study is to develop a new radiomic feature representing the emphysematous density (ED) changes and use their spatial information to characterize the emphysema heterogeneity. The correlational study between lung function and emphysema heterogeneity was conducted to access their statistical relationship. The predictive model was trained using emphysematous density distribution as the predictors and showed strengthen the relationship with lung function. The illustrative examples of emphysema heterogeneity having different impacts on lung function were demonstrated.

## Results

In order to build a predictive model for lung function outcome based on emphysema heterogeneity, we developed a series of methods to extract the anatomical image features of chest CT in the previous works^20^. The spatial information of bullae in the upper and lower lobes can be obtained by using the lung fissure and lobe segmentation method proposed in^21^. The regions of the whole lung are partitioned into upper and lower lobes. The upper lobes include the right upper lobe and the left upper lobe, while the lower lobes include the right middle lobe, the right lower lobe, and the left lower lobe. For the method of approximating the density mask with the best fit to the emphysematous lesion, the binarized CT image uses a single HU threshold. Although Wang *et al*. draws the conclusion of −950 HU being acceptable based on the correlational studies between LAA% and five-category classification (GOLD staging), the HU thresholds corresponding to the highest correlations with FEV1/FVC vary for each lobe, ranging from −925 to −935 and only the right middle lobe has the best result with HU −945. In particular, both upper lobes and lower lobes have the highest correlations with FEV_1_/FVC using HU −935. The proposed method uses single HU −930 to segment the emphysematous regions for the correlational study with FEV_1_/FVC.

### Patient characteristics

**Figure 1:**
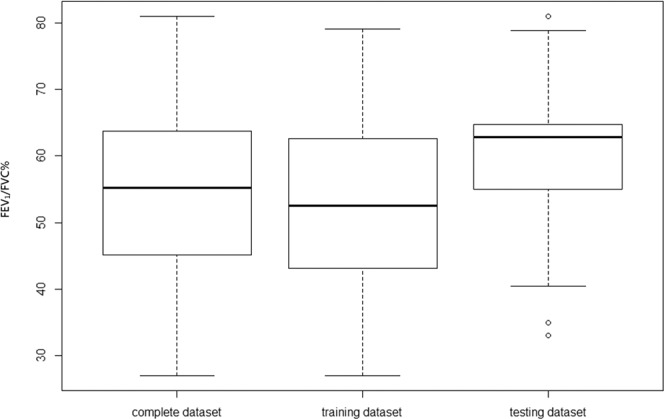
Box plot for each dataset showing both complete and training dataset have nearly normal distribution and equivalent interquartile range. All datasets have median line inside each other’s box.

The study population comprised 99 patients of the complete dataset, was randomly split into 76 subjects of the training dataset and 23 subjects of the testing dataset. Among the complete dataset, 12 subjects have normal functions (FEV_1_/FVC % > 70), showing no or mild emphysema in CT images. The boxplot in Fig. 1 shows that the distribution of FEV_1_/FVC % is balanced in both the complete dataset and training dataset. In patients with FEV_1_/FVC (%) < 70%, the Global Initiative for Chronic Obstructive Lung Disease (GOLD) guideline uses FEV_1_ (% pred.) to classify their COPD severity into four stages ranging from mild to very severe (stage 1, mild: FEV_1_% predicted ≥80%; 2, moderate: 50~70%; 3, severe: 30~49%; 4, very severe: <30%)^22^. The involved subject demographics are summarized in Table 1. The initial correlation study is conducted to select the measurement of PFT as the predicting target. Although the GOLD classification proposes a COPD grading system, the categorical staging result has a weak correlation with LAV%. Instead, both FEV_1_ (% pred.) and FEV_1_/FVC (%) were used in many correlation studies with LAV%. In this study, the complete dataset was evaluated for the correlation study of the disease, and the predictive model derived from the training dataset will be used to predict the outcome of the testing dataset. For example, the correlation coefficients of FEV_1_ (% pred.) and FEV_1_/FVC (%) was studied using the complete dataset to select the one with the strongest relation to LAV%.

**Table 1:** Subject Demographics (n = 99).

Parameter	Mean (±std) or count (%)
Sex male	97 (97.97)
Age	67.3 (12.94)
Height (cm)	166.96 (6.77)
Weight (kg)	67.22 (12.94)
FEV1/FVC %	54.82 (12.74)
FEV1% predicted	66.77 (22.28)
**COPD Severity**	
None	12 (12.12)
GOLD stage I	16 (16.16)
GOLD stage II	48 (48.48)
GOLD stage III	21 (21.21)
GOLD stage IV	2 (2.02)

Abbreviation: FEV1 – forced expiratory volume in one second, FVC – functional vital capacity, GOLD – the global imitative for chronic obstructive pulmonary disease.

**Figure 2:**
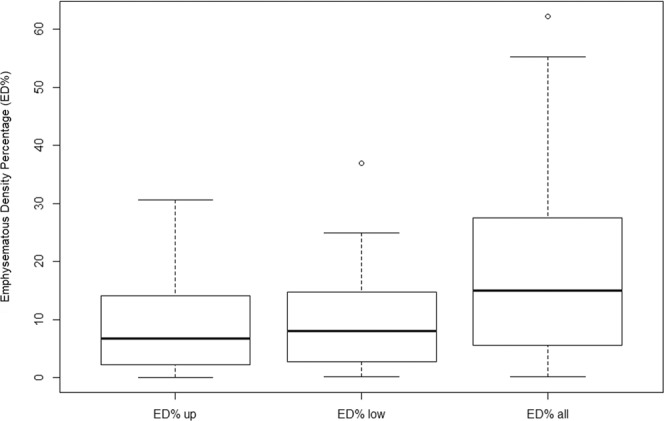
Data distributions among variables of complete dataset (n = 99).

**Figure 3:**
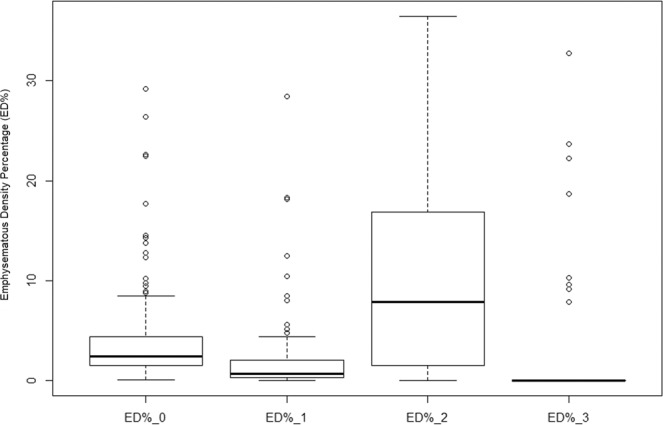
Data distributions among variables of complete dataset (n = 99).

**Figure 4:**
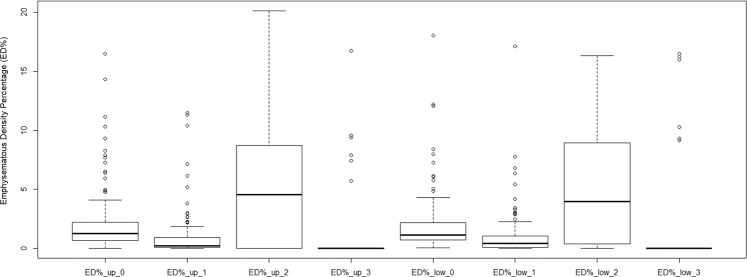
Data distributions among four categories of ED% in upper and lower lobes of complete dataset (n = 99).

While the FEV1/FVC % and FEV1 (% pred.) of the complete dataset have a normal distribution, the studied variables of lobe-based ED% and scale-based ED% do not have corresponding data distribution. As the boxplots of Figs. 2–4 have all shown right-skewed distribution, their individual Pearson’s correlations with lung functions are expected to be weak.fr

### Correlation study between lung functions and LAV%

**Figure 5:**
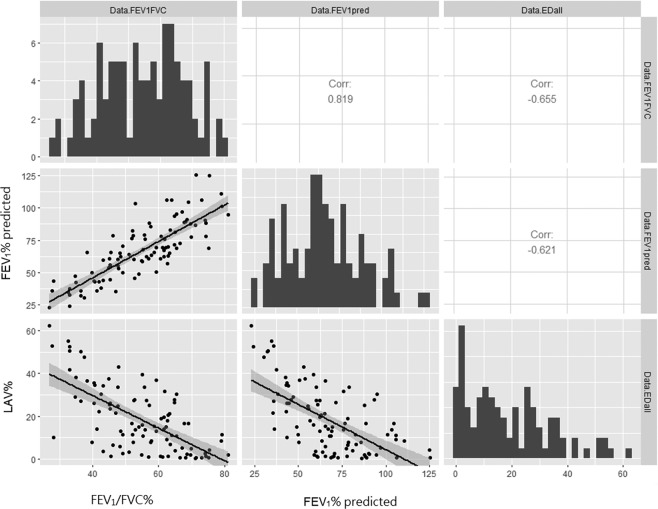
Scatter plot and linear regression of 99 subjects showing the relationship between lung function (FEV_1_/FVC% and FEV_1_% predicted) and LAV%.

From the previous work and related studies, we realize that emphysematous lesions evaluated by LAV% play an important role in correlation with PFT and the progression of emphysema. In order to fit the predictive modeling, we need to determine the most predictable outcome of the lung function correlating to image features in CT images. We first analyze the correlation between LAV% and FEV_1_ (% pred.) and FEV_1_/FVC (%). We also divide the LAV% into upper lobes and lower lobes. The results of comparing Pearson’s correlation are shown in Table 2 and Fig. 5. FEV1/FVC has a higher correlation than FEV1 (% pred.) with LAV% in upper lobes, lower lobes, and whole lung. Hence, we use FEV_1_/FVC % for this study.

**Table 2:** Pearson’s r between LAV% and lung functions of complete dataset (n = 99).

	FEV1 (% pred.)	FEV1/FVC (%)
Upper lobes	P < 0.001 −0.59	P < 0.001 −0.60
Lower lobes	P < 0.001 −0.61	P < 0.001 −0.65
Whole lung	P < 0.001 −0.62	P < 0.001 −0.66

### Emphysema representation

The input for representing the emphysematous lesions is the binarized data masked by a single threshold of HU −930. This input is the same as that of computing LAV%. Applying iterative morphological erosion to the initially connected components of binarized input, we will find multiple local maxima for each connected emphysematous lesion in the same fashion as finding multiple local maxima for the topography map. In general, the lowest contour line encircling the boundary of local maximum separates it from surrounding local maxima. We account for each local maximum as one bulla and the number of connected binarized voxels enclosed by the boundary as the volume of that bulla. The representation of the bulla is the sphere in the size of equivalent volume. One may note that the size of the sphere or its coverage is not the same as the boundary of the emphysematous lesion in the image. Therefore, the morphometric meaning of the sphere is not the same as the results of fitting bullous regions with gaussian mixture models in our previous work^20^. On the contrary, the volume of the sphere is the density of the bulla. The details of the method for clustering emphysematous lesion will be described in the section of the material and method. The hypothesis of representing bulla with volumetric mass density instead of fitting the bullous region with Gaussian kernel will be elaborate in the section of the discussion.

**Figure 6:**
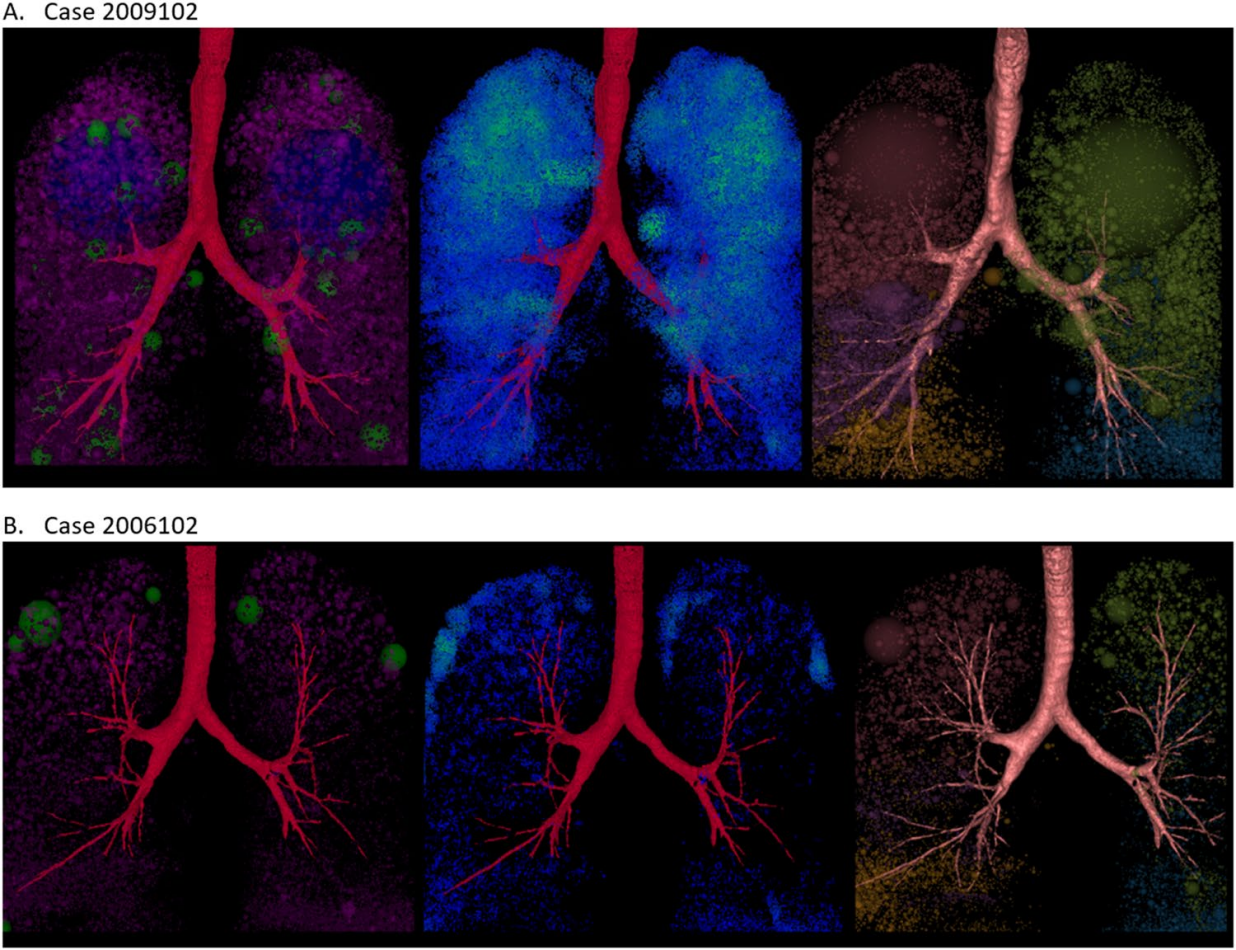
Emphysema heterogeneity with different representations for two cases (**A**,**B**). First column shows emphysematous density (ED) distribution of our approach. Second column shows voxel-wise density map. The third column shows low attenuation clusters (LACs) using commercial quantitative imaging software (Apollo; VIDA Diagnostics, Coralville, IA, USA).

In Fig. 6. We show three different representations of emphysematous lesions in two cases of CT images. Each sphere of emphysematous density (ED) showing in the first column represents an isolated bulla with the number of connected bulla voxels (HU < −930) equivalent to the spherical volume. Each ED can be color-coded according to four categories of scale (small: pink, medium: green, large: blue, and extra-large: yellow). The second column shows the voxel-based density map, such that each bulla voxel counts the number of bulla voxels within the fixed distance, then divides the number by the volume of searching distance to get the density. In the figures, the density is represented by the color gradient from blue to green, such that the blue voxel has lower emphysematous density than a green voxel. Identifying the locations of more condensed bulla voxels, the results of column one corresponds to that of column two. The third columns are the low attenuation clusters (LACs) obtained by using commercial quantitative imaging software (Apollo; VIDA Diagnostics, Coralville, IA, USA). Although the algorithm integrated in the software is not yet clear, a visual inspection shows that they share similar results. However, the advantage of our approach is that the total volume of the ED is the same as the total volume of LAV. The lung functions and LAV% of each case are summarized in Table 3.

**Table 3:** The lung functions and LAV% of each case in Figure 6.

Case	A. 2009102	B. 2006102
Whole lung LAV%	14.74	1.56
Upper lobes LAV%	11.81	0.98
Lower Lobes LAV%	2.93	0.58
FEV1/FVC%	45.23	61.27
FEV1% predicted	55.88	69.37
GOLD stage	3	2

**Figure 7:**
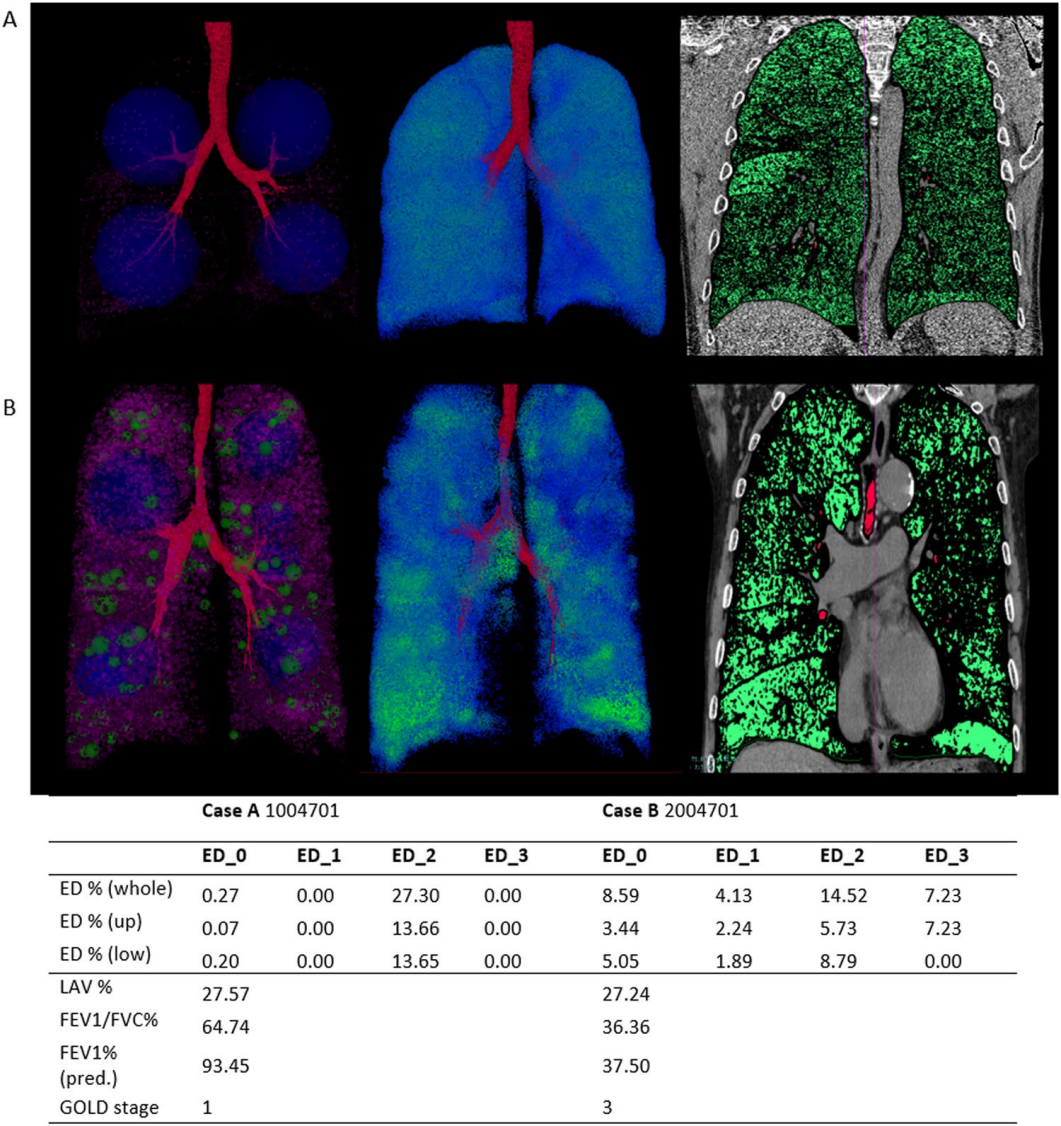
Two cases, **A** and **B** have equivalent LAV%, but also have very different measurements in lung function.

Using the same criteria derived in the previous work to categorize the spheres into four density scales (radius: <5 mm, 5~20 mm, 20~50 mm and >50 mm), the emphysematous density percentage (ED%) is the total volume of spheres in the same category divided by total lung volume. After visually inspecting another two cases (A, B) in Fig. 7 showing different outcomes in lung functions but similar in LAV%, the radiologists raise an intriguing question about their contradicting discrepancy: the possibilities of emphysematous heterogeneity having effects on lung functions. Case A has moderate but homogeneous emphysema occupying the entire lung, whereas case B has heterogeneous emphysema with the composition of various ED%. Case B has more severe lung function than Case A.

### Correlation study between lung function and ED %

In order to evaluate the effects of the density and location of emphysema on the severity of lung function, we first did the pairwise Pearson’s correlation comparison with FEV/FVC. The results are summarized in Table 4. Unlike the results of stronger correlation taking place in LAV% of lower lobes as summarized in Table 2, the correlation between FEV1/FVC and ED does not show a significant difference in different lobes, but in different density scales.

**Table 4:** Pearson’s r between Emphysematous Density (%) and FEV1/FVC (%) (n = 99).

	<5 mm	5~20 mm	20~50 mm	>50 mm
Upper lobe	P < 0.001 −0.53	P < 0.001 −0.51	P < 0.001 −0.32	P < 0.05 −0.22
Lower lobe	P < 0.001 −0.58	P < 0.001 −0.47	P < 0.001 −0.37	P = 0.1 −0.15
Whole lung	P < 0.001 −0.58	P < 0.001 −0.51	P < 0.001 −0.36	P < 0.05 −0.20

### Linear model fitting and derivation of the predictive model

Next, the model derivation and validation were based on the performance of several multiple linear regression models. In this study, different combinations of ED scales and lobar locations were composed to describe the emphysematous heterogeneity (ie. percentages of each ED scales in the upper and lower lobes labeled as ED_up_(0, 1, 2, 3) and ED_low_(0, 1, 2, 3), total percentages of small and large ED scales in both upper and lower lobes, labeled as ED_(all, up, low)_(small, large), and total percentages of ED scales in upper and lower lobes, labeled as ED_up, and ED_low). ED_small is the sum of ED_0 and ED_1 and ED_large is the sum of ED_2 and ED_3. Each variable is defined in the section of the material and method.

In order to find out which of multiple linear regression models is the best fit for the dataset, we systematically derived the models as listed in Table 5. All models were evaluated and selected by Akaike information criterion (AIC) and the difference between Model C and D was evaluated using ANOVA. All the variables in each model were evaluated for collinearity using the variance inflation factor (VIF). No variable of all models has collinearity with others.

**Table 5:** Abbreviation: ED- emphysematous density.

Coefficient estimated (±std) (VIF) p-value	Model-A: Location dependent	Model-B: Size dependent	Model-C Both size and location dependent	Model-D Elaboration of Model-C
Intercept	65.45 (1.58) P < 0.001	64.5 (1.56) P < 0.001	64.73 (1.58) P < 0.001	64.50 (1.58) P < 0.001
ED -up	−0.32 (0.23) (3) P = 0.17			
ED -low	−0.82 (0.24) (3) P < 0.001			
ED-all Small		−0.71 (0.10) (1) P < 0.001		
ED-all Large		−0.43 (0.09) (1) P < 0.001		
ED-low Small			−0.72 (0.39) (3) P < 0.1	−0.720 (0.39) (3) P < 0.1
ED-low large			−0.88 (0.37) (4) P < 0.05	−0.88 (0.37) (4) P < 0.05
ED-up Small			−0.71 (0.38) (3) P < 0.1	−0.77 (0.38) (3) P < 0.05
ED-up Large			−0.02 (0.34) (4) P = 0.96	
ED-up Size-2				−0.09 (0.35) (3) P < 0.80
ED-up Size-3				−0.52 (0.50) (1) P = 0.30
R-squared p-value	0.43 P < 0.001 ***	0.45 P < 0.001***	0.46 P < 0.001***	0.47 P < 0.001***
AIC	739.52	736.83	739.11	739.01

Simple linear regression model estimating outcome of FEV1/FVC % using complete dataset (n = 99).

ANOVA testing Model-C and Model-D: F-value is 1.99, p-value = 0.16.

In order to evaluate the emphysema characteristic on the balanced data, we initially evaluated the linear models on the complete dataset. The Model-A incorporating the lobe dependent ED% which are equivalent to the LAV% in upper lobes and lower lobes. The scale-dependent variables of Model-B having the least AIC values, however, do not have the highest r-squared comparing to other models. Comparing to ED-up of upper lobes, the estimated coefficients of ED_low in the lower lobes are statistically higher, indicating a greater impact on FEV1/FVC due to the emphysematous lesion in the lower lobes. Comparing to the percentage of the highly condensed emphysematous lesion (ED_all_large), the estimated coefficients of ED_all_small is statistically higher, indicating the greater impact of loosely spread emphysematous lesions on FEV1/FVC in this dataset. The contribution of location and density-dependent factors on the lung function can be fluctuated due to the complexities of the disease, for example, the influence of bronchiectasis. However, the strategy of model fitting exploited the statistically significant difference, and furtherly segmented the variables of ED% to formulate Model-C, which is both scale and location dependent. The results of Model-C show mild improvement in performance and induces an insignificant variable of ED% of the highly condensed emphysematous lesion (ED_up_large) in upper lobes, which can be safely eliminated for its particularly low coefficient (0.02, p = 0.96). Model-D is the elaboration of Model-C. Model-D replaced E_up_large with two split scales (E_up_2 and E_up_3) using scale differentiation criteria introduced in the previous work. Model-D has the best performance among the models.

### Prediction performance evaluation

The derived predictive models modeled FEV_1_/FVC% versus ED% of different density scales in upper and lower lobes. The model is trained using the training dataset (n = 75) by 10-fold cross-validation repeated for 30 times after randomization of the order of subjects in each dataset. Based on the results summarized in Table 6, the final model of both Model-C and D shows that the percentage of ED% in the lower lobes is more associative than in the lower lobes on lung function, FEV_1_/FVC%. Although the contributions of the predictors are location and scale-dependent, highly condensed ED% in the upper lobe may not associate with the results of FEV_1_/FVC%.

**Table 6:** Comparing the performance of predictive model between model C and D using training dataset; using linear regression model with 10-fold cross-validation repeated for 30 times.

Coefficients estimated (±std) (VIF) p-value	Model-C Complete dataset (n = 76)	Model-D Training dataset (n = 76)
Intercept	62.58 (1.80) P < 0.001	62.43 (1.82) P < 0.001
ED-low Small	−0.54 (0.43) P = 0.22	−0.53 (0.44) P = 0.22
ED-low large	−0.87 (0.44) P < 0.05	−0.85 (0.41) P < 0.05
ED-up small	−0.74 (0.41) P < 0.1	−0.77 (0.42) P < 0.1
ED-up large	0.00 (0.42) P < 0.99	
ED-up Size-2		0.06 (0.43) P = 0.89
ED-up Size-3		−0.24 (0.58) P = 0.68
R-squared p-value	0.44 P < 0.001 ***	0.44 P < 0.001 ***
AIC	570.24	571.80
**repeated 10-fold cross-validation 30 times**		
mean (±std)		
RMSE	9.72 (2.40)	9.66 (2.33)
R-squared	0.46 (0.21)	0.47 (0.23)
MAE	8.00 (1.99)	8.04 (2.01)

Final linear regression model estimating outcome of FEV1/FVC % n = 76.

Abbreviation: RMSE – root mean square error, MAE –mean absolute error.

**Figure 8:**
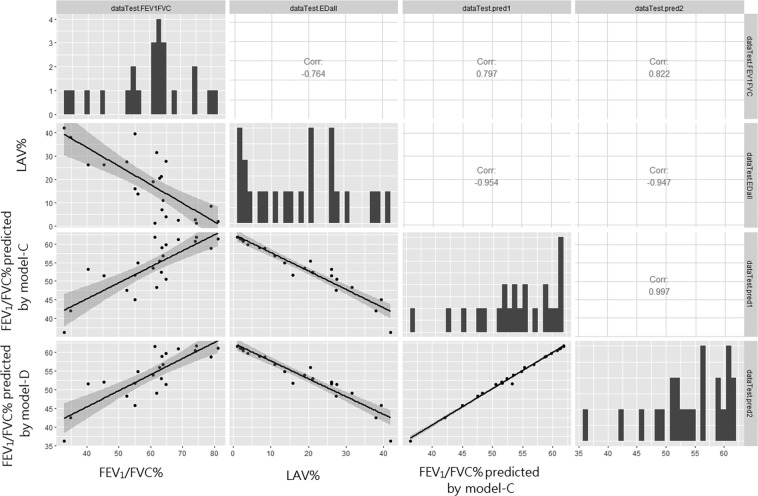
Scatter plots and linear regression of showing the relationship between observed and estimated lung function, FEV_1_/FVC % in predictive model (C and D); and comparing the scatter plots of FEV_1_/FVC versus LAV% in testing dataset.

In contrast to the results using a complete dataset, only highly condensed emphysematous lesions in lower lobes and less condensed emphysematous lesion in upper lobes have statistical significance in the final models of the training data. The RMSE (9.66% ± 2.3) of Model-D is slightly lower than RMSE (9.72 ± 2.40) of Model-C yielding better prediction in the testing dataset. The correlation between the LAV% (labeled as dataTestEDall) of CT and FEV_1_/FVC % of PFT in the testing dataset (n = 23) is −0.76. The correlation coefficient is improved to r = 0.80 (labeled as dataTestpred1) and r = 0.82 (labeled as dataTestpred2) in Model-C and Mode-D respectively (Table 7 and Fig. 8).

**Table 7:** Correlating with lung function, FEV1/FVC on testing dataset (n = 24).

Predictor(s)	LAV %	Predicted by Model-C Both size and location dependent	Predicted by Model-D: Elaboration of Model-C
Pearson’s r	P < 0.001 −0.76	P < 0.001 0.80	P < 0.001 0.82

## Discussion

Extensive works on characterizing the morphology of the parenchymal destruction, such as the Heterogeneity index (slope of emphysema index in upper-lower direction, anterior-posterior direction, and central-peripheral direction)^23^ and power-law analysis^24^ were used to evaluate the treatment outcome of LVR. Coxson *et al*. estimated emphysematous lesion size by the cumulative number of connected low attenuation voxels and used power-law analysis to correlate the number and size of emphysematous lesions with treatment response of lung volume reduction surgery (LVRS)^24^. The result confirms that patients with large upper lobe lesions respond better than patients with smaller but well-distributed bullae. McLennan *et al*. used spherical structure to approximate the connected low attenuation voxels in order to provide the visualization of low attenuation clusters (LACS)^25^. Lee *et al*. took a similar approach and filtered LAV with Gaussian low pass kernel to iteratively searching for the centroid voxels of bullous structures^26^. However, these studies do not correlate the findings with lung functions. Recent studies have examined the lobar distribution of emphysema and found that PFT is different among patients with upper or lower lobe dominant emphysema or homogeneous distribution of emphysema^13,27–29^. Choosing the target lung tissue for LVR in order the remove excessive non-functioning tissue while preserving the healthier tissue was a challenging task. While the emphysema heterogeneity is associated with lung function decline and treatment response in LVR, this study utilizes emphysema heterogeneity to predict regional lung function decline. By examining the association between lung function and a novel index of emphysema heterogeneity, a prediction of lung function decline is derived for identifying target selection in LVR, which the conventional CT emphysema index, such as LAV% cannot be used to detect.

In the result section, we have evaluated several multiple linear regression models and discovered that ED% in the lower lobes has the largest impact on FEV_1_/FVC %. Emphysematous density represents the number of bulla voxels per unit lung volume which is the same definition as LAV%. In order to avoid the confusion with conventional LAV%, we use ED% to represent the emphysematous density percentages of the local maxima within connected components. The total percentage of ED is proved to be the same as LAV%.

The heterogeneous characteristic extracted by the proposed method can be described by two cases in Fig. 7. The last column has a cross-sectioned image with an emphysema lesion marked in green. The second column is the result of voxel-based density in the fixed distance volume. In this example, we use the searching range in the radius of 10 mm to accumulate the number of bulla voxels used for computing the density of each bulla voxel. The voxels on the density map with high value show the locations of hollow spaced emphysematous lesions. The density map of Case A shows a well diffused, distributed bulla voxels occupying the entire lung without any highlighting areas like those shown in Case B. The results of density map correspond to the masked area of cross-sectioned images on the right. Although the result is intuitively simple to visualize the complexity of the disease, it is difficult to quantify the number and the distribution of low attenuation clusters (LACs) attributing to the emphysema heterogeneity using density map.

The cross-sectioned image of Case A shows that the binarized pixels are most likely well distributed across the entire lung with some condensed areas on the top. The results in the first column of Case A show four large blue spheres (color-coded as ED_2) with numerous small spheres surrounding them. These four local maxima represent the well-connected diffused lesions in the right upper, left upper, right lower and left lower lobes. The volume of the sphere is the volume of the bulla voxels connected to the local maxima. The method derived in our previous work, on the other hand, will occupy the entire lung with various small spheres as an image descriptor of bullous regions. The cross-sectioned image of Case B shows multiple isolated or weakly connected but condensed lesions scattering on both sides of the lung. The results of the density map in the second column also correspond with the result by highlighting the locations with bright green voxels. The green spheres have lower ED % (color-coded as ED_1). Both blue and green spheres in the first column have overlapped with the regions of LACS in the density map. Pink sphere has the lowest ED% (color-coded as ED_0). Comparing to the result of four large spheres in Case A, the compositions of spheres in different sizes reveals higher complexity of emphysematous heterogeneity in Case B. In contrast to Case A, the result of Case B has similarities with the result of using Gaussian mixture model (GMM) from previous work. Although both methods utilize the searching of local maxima as the initial spheres, GMM is the result of competing for spaces by fulling the Gaussian distribution of bulla voxels at the location where the sphere is occupying.

**Figure 9:**
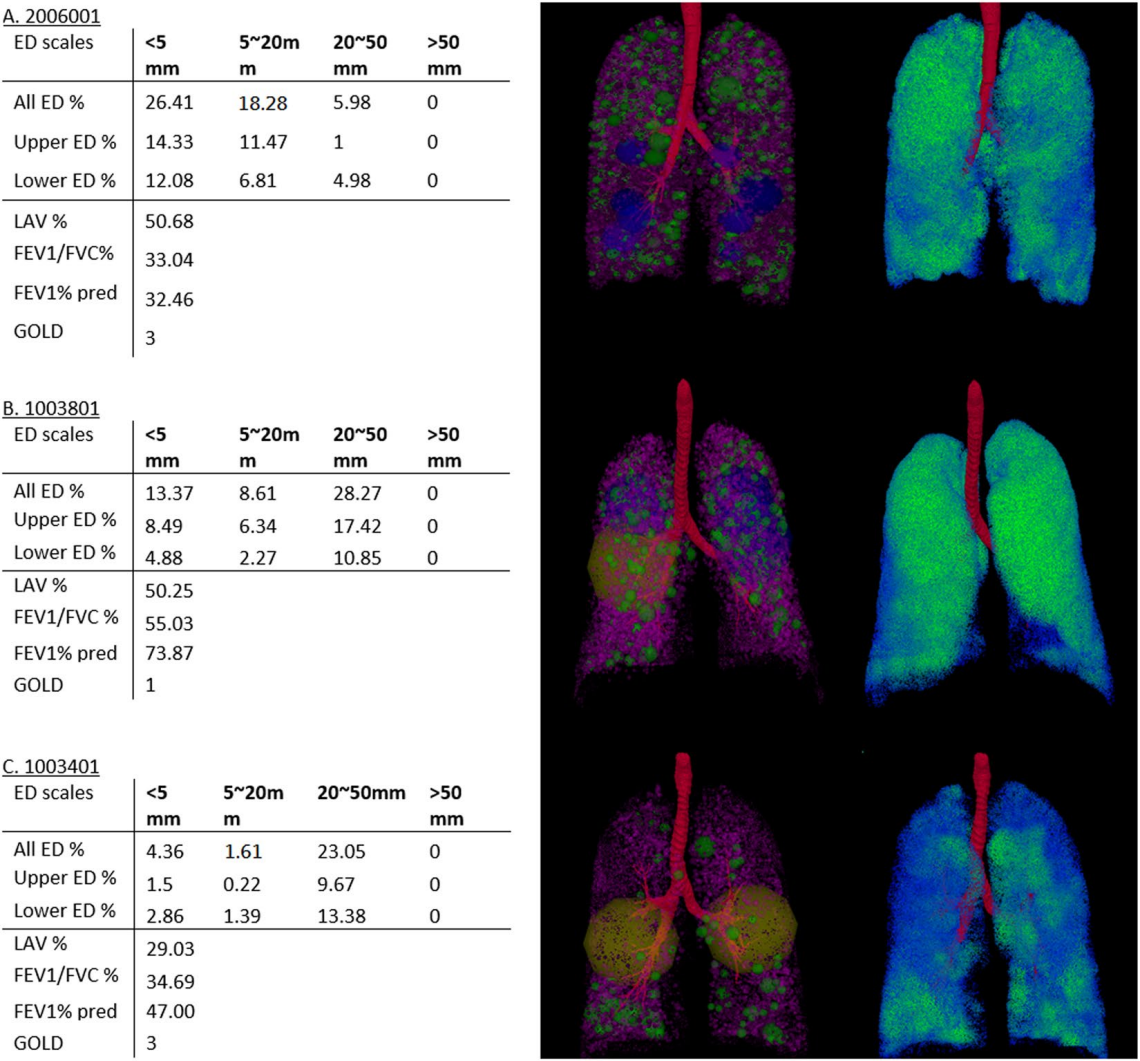
Cases studies of dispersed cases inconsistent to measured FEV_1_/FVC%.

In addition to a 3D morphological descriptor describing emphysema, this study also proposed a predicting model for FEV_1_/FVC using image features obtained from CT images. Having equivalent LAV% of 27%, the predicted FEV_1_/FVC% for Case A is 50.62% comparing to measured FEV_1_/FVC% of 64.74, and the predicted FEV_1_/FVC% for Case B is 43.1% comparing to observed 36.36% using derived the predictive model. The FEV_1_/FVC of Case B is predictively more severe than the Case A. Bullous region detected by our algorithm is the result of completely connected bulla preserving the topological bullous structures. In other words, a large emphysematous lesion (ED-large) found represents a large hollowed space occupied in the lung such that the iterative erosion cannot break it into smaller emphysematous regions. As a result, a larger accumulated volume of ED, has the theoretical meaning of highly condensed emphysematous lesion. Many comparable examples can be found among patients with similar LAV% but a totally different proportion of destruction or vice versa (Fig. 9).

To study the association of emphysema heterogeneity with FEV1/FVC, we compare the results of the predicted value of FEV1/FVC% and LAV% with measured FEV1/FVC%. In the study of 87 mild to very severe COPD patients and 12 normal control subjects, we found the inconsistent correlation between FEV1/FVC% and LAV% in some cases while other studies have proven that LAV% being useful in characterizing phenotypes of emphysema. After the observational study of inconsistent cases such as those shown in Figs. 7 and 9, we found that the heterogeneity of emphysematous can be expressed as the compositions of emphysematous density on different scales. Although the predictive model of this study does not induce the cause and effect between the emphysema heterogeneity and FEV1/FVC%, a significantly higher correlation with LAV% in the lower lobes requires further investigation in the future work. Smaller isolated emphysematous lesions which also have a higher correlation than those with higher ED% in Model-B also suggest that the impact of decreasing predicted FEV1/FVC%. Smaller isolated lesions have the volume of bulla voxels equivalent to the radius less than 20 mm in this study. In the derived Model-C and Model-D used for prediction, the total estimated coefficients of small ED% in both upper and lower upper lobes contributed more than half of the negative impact (ie. 1.6 of 2.98 in Model-D).

In addition to the predictive model, the ED% representation can be a visualization technique for assessing the distribution and composition of bullae in terms of their connected volume and positions. During the training process of using randomly selected 76 subjects from the complete dataset, the information on the location of the bullous area contributes to the severity of lung function as shown on the summary of final models in Table 5. For example, Case A in Fig. 7 has relatively better FEV1/FVC than Case B, but the total LAV% is considerably high, particularly, the percentage of ED_2 according to the clinical standard. The predictive model-D has a coefficient of −0.09 for ED_2 in the upper lobes to adjust the impact of high LAV% on the FEV1/FVC in upper lobes. Both Case A and B in Fig. 9 have emphysematous lesion taking over half of the lung (LAV% > 50) and however have different severity in their PFTs. Case A is diagnosed with severe COPD of GOLD stage 3 (FEV1/FVC 33.04% and FEV1 predicted 32.46%). Case B with equivalent LAV% to Case A has the FEV1/FVC 55.03% and predicted FEV1 26.65%. Although they both have equivalent LAV%, the FEV1/FVC measured by PFTs is 33.04% in Case A and 55.03% in Case B. The predictive model estimates the predicted FEV1/FVC 26.65% in Case A and 36.8% in Case B. Hence, estimated results also show that Case A has more severe lung dysfunction than Case B. On the other hand, both Case A and C have equivalent FEV1/FVC % measured by PFTs. Case C, however, has shown less emphysematous destruction (LAV% 29.03) as compared to Case A in CT images. Although the predicted lung function of Case B is only 10% of FEV1/FVC better than Case A, the compositions of ED is much more complicated in Case A. The visual inspection of Case C has shown greater heterogeneity comparing to both Case A and B, leading to the greater decline in FEV1/FVC %. The results show that the estimated lung function can be accessed visually using the proposed representation.

Related studies suggest the negative impact of air-trapping due to the emphysema^30^. The future work will apply the same method on the air-trapping of HU < −856 in expiration CT to study their dynamic interaction. The quantitative evaluation of emphysema heterogeneity is limited to the multiple linear regression modeling using the total percentage of ED in different scales and locations without taking the spatial complexity into consideration. The future work can incorporate a higher level of interaction between variables, such as the clustering of ED in mixed scales. Another future work is to employ multilevel modeling due to the hierarchical relationship in inspiration and expiration CT images and patients to medical centers as we have discovered the variance in patients from different medical centers.

In conclusion, the contribution of developed ED representation allows us to understand the emphysema heterogeneity and their association with lung function based on lesion size and location, whereas a single mean density value of LAV% cannot assess the morphological complexities of the disease^30,31^. Our findings indicate that smaller isolated lesions have a greater negative impact than larger condensed or homogenously diffused lesions on FEV_1_/FVC%, and comparatively greater FEV_1_/FVC% decline is due to the lesions located in the lower lobes. The proposed representation of ED% can also be applied to the predictive modeling of FEV_1_/FVC% to provide an additional preoperative evaluation for LVR. The voxel-wise prediction model of airflow limitation in the regions with emphysema destruction was not available in the previous studies. Utilizing the derived model, the functional evaluation can be assigned to designated regions, particularly the most “functionally affected” tissue for LVR treatment approach and avoid less diseased portions of the lobe.

## Materials and Methods

### Medical research ethics and image source

This retrospective study was approved by the institutional review board (IRB) of the National Taiwan University Hospital (NTUH) and Cardinal Tien Hospital (CTH). The volumetric CT scans were taken at full inspiration. A total of 87 subjects with symptoms of chronic obstructive pulmonary disease (GOLD stage 1~4) and 12 normal subjects was included; 55 cases of NTUH, and 44 cases of CTH. Subject demographics are summarized in Table 1. All subjects are evaluated in the correlational studies and 76 of randomly selected subjects are trained in the predictive modeling for testing on the remaining 23 subjects.

### Airway segmentation and lung parenchyma segmentation

In this study of using three-dimensional CT to reconstruct the anatomical structures of the lung, we utilize the in-house algorithm to extract the airway based on the multi-angle probing approach and to segment the lung region based on the adjustable rolling ball approach. The multi-angle probing tracheal extraction algorithm is to first set the initial position of the tracheal section at the top of the lung and then obtain the initial tracheal lumen by the region growing method, and then to obtain the centerline by the thinning algorithm. The tree-like structure of the trachea is constructed through the center line, and the endpoint of the tree is used as the starting point for the downward extension. At various branch points, the multi-directional area is searched for the area that may extend downward, and the search of constrained areas is continuously repeated until there is no tubular structure obtained. Firstly, the region growing algorithm is applied to the opening of the trachea at a lower threshold to obtain an entire lung including an airway. The lung volume is then deducted from the airway volume to divided into two lobes. After filling the holes in the lobes with the rolling ball algorithm, the outer area of the lung region can be found. Then, using the rib information as the constraint and the size estimation of the three-dimensional adjustable rolling ball, the method can segment the lung region into the left and right lobe.

### Modeling of the emphysematous lesion using the local maxima of bulla voxels

In order to develop a scale-dependent descriptor featuring the distribution of emphysematous density, which also plays an important role in the restriction of elastic recoil and radial traction, we proposed an algorithm to search for the positions of the LACs while preserving the values of their emphysematous density. By applying iterative erosion to the binarized image data, we will collect the isolated bullous voxels and mark them as the local maxima of its surrounding bulla voxels. Each local maximum will accumulate the number of eroded voxels from previous steps of erosion.

To find the corresponding clusters of the emphysematous lesions in the low attenuated volume, the input binarized image is the mask using a fixed HU threshold value. Then we will apply the binarized image of connected components to recursive processing of 3D erosion using the radius of 3 voxels. During image processing, the algorithm also records the number of erosions until the disappearance of each connected component. The result of the procedure is a 3D contour map with isolated local maxima, where the voxels are eroded the last without other attaching voxels. We name the eroded regions of each iteration as enclosed regions of *R*. Each region (*R*) enclosed by contour line has a number (*n*) of voxels (*s*) and each voxel is a subset of the region: *R* = {*s*_1_*, s*_2_*,… s*_*n*_}. Each subset of *R* has two attributes, the weight (*w*) and the iterative order (*e*) of the erosion, namely *s*_*(w, e)*_. We start the workflow of searching for local maxima by giving initial condition *e* = 1*, w* = *1* for each subset, ie. *s*_*(1, 1)*_. During the erosion, for each subset of a certain region, (*s*′ *of* *R*′) we check for two conditions.

1. If *s*′ has attaching subset of another region (*s*″ *of* *R*″) with edge greater than 0 and *e(s* ″*)* > *e(s*′*)*, then the region s′ belongs to its attaching to another region that is eroded later, and *R’* cannot be the local maxima.
2. If s′ has attaching subset of another region (*s*″ *of* *R*″) with edge small than its edge: *e(s* ″*)* < *e(s*′*)*, then we can merge them by assigning *w(s*′*)* = *w(s*′*)* + *w(s*″*)* and *w(s*″*)* and *e(s*″*)* = 0.

Eventually, each local maximum has only one voxel, *s*_*(w, e)*_ left with the highest iterative order, *e* and most accumulated weight, *w*. The weight of the local maxima is equivalent to the enclosed volume of the bulla voxels and the initial position of the last voxel is close to the center of the emphysematous lesion in the image. The center of the lesion can be reallocated by calculating the Gaussian distribution of bulla voxels belonging to the local maximum of the connected component.

An emphysematous region of loosely connected bulla regions can have multiple local maxima identified during the procedure. A fully connected component, on the other hand, may only have one local maximum. Now, we have a list of local maxima with a number of voxels attaching. In order to plot them in the image, we use a sphere with a radius of volume equivalent to *w(s)*. ED% is the percentage of *w(s)* over the entire lung volume. The result of emphysema representation is the transformation of low attenuation volume (LAV) into emphysematous density (ED). Their total percentages in the lung volume are the same. We then classify the scales of ED% into four size groups like those defined in^32,33^.

Two emphysematous lesions may have the same size in numbers of bulla voxels, but the more condensed, fully connected region occupies a smaller proportion of the lung while the loosely connected region having an enormous number of local maxima can easily occupy the entire lung^32^.

### Statements

All experiments and methods were performed in accordance with relevant guidelines and regulations. All experimental protocols were approved by a named institutional committee. Specifically, the retrospective study of CT imaging and PFT were approved by the Institutional Review Board (IRB) and the Research Ethics Committee (REC) at NTUH and CTH. Informed consent was obtained from all subjects, and all methods were carried out in accordance with the relevant guidelines and regulations of IRB/REC.

## Supplementary information

Data supplement

testing dataset and training dataset

## Data Availability

The supplementary datasets generated during and/or analyzed during the current study are available. The CT data are available from the corresponding author on a reasonable request.
