# Supplementary material for: Predictive Modelling of Lung Function using Emphysematous Density Distribution
Source: Sci Rep. 2019 Dec 24;9:19763. doi: 10.1038/s41598-019-56351-9 (PMC6930211; doi:10.1038/s41598-019-56351-9)
Supplement: Supplementary file 1 — Data supplement [file 41598_2019_56351_MOESM1_ESM.docx]

**Predictive Modelling of Lung Function using**

**Emphysematous Density Distribution**

Kuo-Lung Lor, Cheng-Pei Liu, Yeun-Chung Chang, Chong-Jen Yu, Cheng-Yi Wang, Ming-Jui Chung, Fan-Ya Lin, Chung-Ming Chen

DATA SUPPLEMENT

DataTest. Supplemental dataset for testing the predictive model

|  | UID | Age | Height  (cm) | Weight  (kg) | Sex | BMI | EDup | EDlow | EDall | EDup0 | EDup1 | EDup2 | EDup3 | EDlow0 | EDlow1 | EDlow2 | EDlow3 | FEV1FVC | FEV1pred | gold |
| --- | --- | --- | --- | --- | --- | --- | --- | --- | --- | --- | --- | --- | --- | --- | --- | --- | --- | --- | --- | --- |
| 1 | 1000201 | 84 | 163.2 | 58 | m | 21.77648 | 10.08 | 17.33 | 27.41 | 0.9 | 0.17 | 9.01 | 0 | 2.2 | 0.24 | 14.88 | 0 | 52.55814 | 60.10638 | 2 |
| 2 | 1002901 | 66 | 164.2 | 57.9 | m | 21.47495 | 14.54 | 11.58 | 26.13 | 2.56 | 0.21 | 11.77 | 0 | 2.04 | 0.99 | 8.55 | 0 | 45.37815 | 44.81328 | 3 |
| 3 | 1003101 | 67 | 164 | 101.5 | m | 37.73795 | 12.42 | 8.09 | 20.51 | 0.51 | 0 | 11.91 | 0 | 0.43 | 0 | 7.66 | 0 | 62.8692 | 62.8692 | 2 |
| 4 | 1004101 | 47 | 178 | 70.8 | m | 22.34566 | 3.87 | 3.08 | 6.95 | 1.69 | 0.17 | 2.01 | 0 | 1.12 | 0.11 | 1.85 | 0 | 63.57759 | 82.86517 | 1 |
| 5 | 1004701 | 52 | 163.3 | 63 | m | 23.6248 | 13.73 | 13.86 | 27.59 | 0.07 | 0 | 13.66 | 0 | 0.2 | 0 | 13.65 | 0 | 64.73552 | 93.45455 | 1 |
| 6 | 1004801 | 77 | 166 | 70 | m | 25.40282 | 9.65 | 9.27 | 18.92 | 1.87 | 0.21 | 7.57 | 0 | 1.37 | 0.06 | 7.84 | 0 | 60.77739 | 78.18182 | 2 |
| 7 | 1005201 | 73 | 161.6 | 65 | m | 24.89033 | 17.34 | 14.22 | 31.56 | 2.59 | 0.87 | 13.88 | 0 | 1.66 | 0.1 | 12.45 | 0 | 61.8677 | 75.71429 | 2 |
| 8 | 1007001 | 51 | 172.1 | 69 | m | 23.29632 | 10.09 | 11.07 | 21.16 | 0.81 | 0.36 | 8.93 | 0 | 0.83 | 0.04 | 10.2 | 0 | 63.30275 | 65.09434 | 2 |
| 9 | 1007701 | 67 | 164 | 101.5 | m | 37.73795 | 12.42 | 8.09 | 20.51 | 0.51 | 0 | 11.91 | 0 | 0.43 | 0 | 7.66 | 0 | 62.8692 | 62.8692 | 2 |
| 10 | 1009401 | 81 | 179 | 66.6 | m | 20.78587 | 22.56 | 16.88 | 39.44 | 4.97 | 1.44 | 16.15 | 0 | 3.95 | 1.51 | 11.42 | 0 | 55.04202 | 58.48214 | 2 |
| 11 | 1010301 | 74 | 165 | 64 | m | 23.50781 | 16.73 | 9.39 | 26.12 | 2.27 | 0.4 | 6.19 | 7.88 | 1.62 | 0.66 | 7.11 | 0 | 40.41667 | 43.49776 | 3 |
| 12 | 1010401 | 69 | 162.2 | 63.4 | m | 24.09836 | 4.91 | 11 | 15.91 | 1.44 | 0.71 | 2.76 | 0 | 0.52 | 0.03 | 10.44 | 0 | 55.04202 | 58.48214 | 2 |
| 13 | 2000901 | 70 | 158 | 68 | m | 27.23922 | 15.99 | 22.05 | 38.04 | 4.76 | 3.03 | 8.2 | 0 | 8 | 5.42 | 8.61 | 0 | 34.87395 | 42.5641 | 3 |
| 14 | 2001201 | 79 | 168 | 58 | m | 20.54989 | 7.15 | 6.52 | 13.67 | 2.14 | 2.69 | 2.32 | 0 | 2.18 | 2.49 | 1.85 | 0 | 55.97484 | 75.74468 | 2 |
| 15 | 2001701 | 79 | 165 | 63 | m | 23.1405 | 0.78 | 1.55 | 2.33 | 0.75 | 0.03 | 0 | 0 | 0.67 | 0.88 | 0 | 0 | 68.66197 | 90.69767 | 1 |
| 16 | 2004201 | 71 | 164 | 48 | m | 17.84652 | 18.64 | 23.26 | 41.91 | 10.31 | 6.14 | 2.2 | 0 | 12.19 | 6.37 | 4.68 | 0 | 32.95455 | 37.5 | 3 |
| 17 | 2006101 | 59 | 174 | 72 | m | 23.78121 | 0.94 | 0.13 | 1.07 | 0.75 | 0.19 | 0 | 0 | 0.13 | 0 | 0 | 0 | 61.27321 | 69.36937 | 2 |
| 18 | 2006501 | 70 | 162 | 48 | m | 18.28989 | 4.2 | 6.77 | 10.97 | 1.88 | 0.6 | 1.71 | 0 | 3.02 | 2.94 | 0.8 | 0 | 63.93443 | 105.8824 | 1 |
| 19 | 2009501 | 66 | 165 | 74 | m | 27.1809 | 1.35 | 2.51 | 3.85 | 1.18 | 0.17 | 0 | 0 | 1.09 | 0.28 | 1.13 | 0 | 64.87342 | 81.02767 | 1 |
| 20 | 1007401 | 52 | 167.9 | 85.3 | m | 30.25852 | 4.89 | 3.41 | 8.3 | 1.87 | 0.09 | 2.93 | 0 | 1.12 | 0.7 | 1.59 | 0 | 78.89273 | 110.6796 | 0 |
| 21 | 2003401 | 74 | 158 | 60 | m | 24.03461 | 1.99 | 0.61 | 2.6 | 1.56 | 0.43 | 0 | 0 | 0.39 | 0.21 | 0 | 0 | 73.99103 | 90.16393 | 0 |
| 22 | 2005801 | 85 | 152 | 44 | f | 19.04432 | 0.4 | 0.83 | 1.23 | 0.16 | 0.24 | 0 | 0 | 0.38 | 0.45 | 0 | 0 | 74.34211 | 77.93103 | 0 |
| 23 | 2008501 | 49 | 174 | 81 | m | 26.75386 | 1.02 | 0.83 | 1.85 | 0.73 | 0.29 | 0 | 0 | 0.62 | 0.21 | 0 | 0 | 81.08747 | 94.49036 | 0 |

DataTrain. Supplemental dataset for training the predictive model

|  | UID |  | Age | Height  (cm) | Weight  (kg) | Sex | BMI | EDup | EDlow | EDall | EDup0 | EDup1 | EDup2 | EDup3 | EDlow0 | EDlow1 | EDlow2 | EDlow3 | FEV1FVC | FEV1pred | gold |
| --- | --- | --- | --- | --- | --- | --- | --- | --- | --- | --- | --- | --- | --- | --- | --- | --- | --- | --- | --- | --- | --- |
| 1 | 1002401 |  | 55 | 168.4 | 64.6 | m | 22.77972 | 6.6 | 7.25 | 13.85 | 1.2 | 0.1 | 5.31 | 0 | 1.01 | 0.41 | 5.82 | 0 | 41.17647 | 36.2069 | 3 |
| 2 | 1002701 |  | 57 | 169.2 | 56.6 | m | 19.77041 | 5.45 | 5.19 | 10.63 | 1.43 | 0.21 | 3.8 | 0 | 1.14 | 0.07 | 3.98 | 0 | 65.34653 | 68.75 | 2 |
| 3 | 1003301 |  | 76 | 163.5 | 64.3 | m | 24.05334 | 14.13 | 10.61 | 24.74 | 0.84 | 0 | 13.3 | 0 | 0.86 | 0.01 | 9.73 | 0 | 47.56098 | 55.45024 | 2 |
| 4 | 1003401 |  | 83 | 165.3 | 49.3 | m | 18.04269 | 11.39 | 17.64 | 29.03 | 1.5 | 0.22 | 9.67 | 0 | 2.86 | 1.39 | 13.38 | 0 | 34.68635 | 47 | 3 |
| 5 | 1003501 |  | 61 | 172 | 72 | m | 24.33748 | 26.36 | 24.05 | 50.41 | 4.88 | 2.66 | 10.91 | 7.9 | 4.87 | 2.93 | 16.24 | 0 | 36.32813 | 32.06897 | 3 |
| 6 | 1003601 |  | 63 | 157.2 | 54.4 | m | 22.01374 | 27.78 | 15.37 | 43.16 | 6.39 | 2.25 | 19.14 | 0 | 3.08 | 1.75 | 10.54 | 0 | 47.91667 | 52.99539 | 2 |
| 7 | 1003701 |  | 73 | 158 | 56 | m | 22.4323 | 27.96 | 24.56 | 52.52 | 7.67 | 5.19 | 15.09 | 0 | 6.14 | 2.87 | 15.55 | 0 | 33.09859 | 24.2268 | 4 |
| 8 | 1003901 |  | 75 | 179.2 | 72.5 | m | 22.57678 | 17.28 | 17.18 | 34.46 | 0.54 | 0 | 0 | 16.74 | 1.07 | 0.1 | 0 | 16.01 | 40.72848 | 43.00699 | 3 |
| 9 | 1004001 |  | 68 | 171.6 | 84.4 | m | 28.66209 | 18.61 | 15.53 | 34.15 | 2.29 | 0.27 | 6.47 | 9.58 | 2.14 | 0.49 | 12.9 | 0 | 43.11594 | 44.07407 | 3 |
| 10 | 1004401 |  | 63 | 170 | 70 | m | 24.22145 | 7.34 | 5.53 | 12.88 | 1.42 | 0.1 | 5.83 | 0 | 0.98 | 0.13 | 4.42 | 0 | 59.31677 | 69.2029 | 2 |
| 11 | 1004501 |  | 60 | 159.9 | 45.5 | m | 17.79568 | 24.77 | 22.83 | 47.59 | 6.53 | 1.52 | 16.72 | 0 | 5.78 | 1.11 | 15.94 | 0 | 37.5 | 30.37975 | 3 |
| 12 | 1004601 |  | 62 | 161.7 | 55.9 | m | 21.37922 | 23.36 | 14.06 | 37.41 | 5.94 | 1.72 | 15.7 | 0 | 2.88 | 0.24 | 10.93 | 0 | 44.79167 | 53.75 | 2 |
| 13 | 1005001 |  | 54 | 176.8 | 83.7 | m | 26.77694 | 7.79 | 6.35 | 14.13 | 1 | 0.08 | 6.71 | 0 | 0.91 | 0.06 | 5.36 | 0 | 61.66667 | 67.06949 | 2 |
| 14 | 1005101 |  | 63 | 166.6 | 61.9 | m | 22.30184 | 16 | 17.59 | 33.59 | 1.57 | 0.17 | 6.84 | 7.42 | 1.2 | 0.16 | 0 | 16.23 | 47.57576 | 60.38462 | 2 |
| 15 | 1005301 |  | 65 | 172.1 | 78 | m | 26.33497 | 6.15 | 10.37 | 16.52 | 0.98 | 0.09 | 5.09 | 0 | 0.7 | 0.05 | 9.61 | 0 | 67.75956 | 88.57143 | 1 |
| 16 | 1005401 |  | 63 | 162.1 | 81.5 | m | 31.01641 | 6.47 | 8.48 | 14.95 | 1.1 | 0.13 | 5.25 | 0 | 1.04 | 0.11 | 7.32 | 0 | 53.91705 | 48.95397 | 3 |
| 17 | 1005501 |  | 76 | 174.3 | 80 | m | 26.33269 | 11.92 | 15.37 | 27.29 | 0.68 | 0.19 | 11.06 | 0 | 1.73 | 0.25 | 3.13 | 10.26 | 56.25 | 62.30769 | 2 |
| 18 | 1005601 |  | 66 | 171 | 58 | m | 19.83516 | 12.19 | 18.27 | 30.46 | 2.44 | 1.23 | 8.52 | 0 | 4.09 | 2.94 | 11.22 | 0 | 42.62295 | 38.23529 | 3 |
| 19 | 1005701 |  | 77 | 165 | 70.1 | m | 25.74839 | 6.62 | 6.5 | 13.12 | 1.42 | 0.06 | 5.14 | 0 | 0.98 | 0.33 | 5.18 | 0 | 48.37545 | 62.32558 | 2 |
| 20 | 1005901 |  | 57 | 170 | 70 | m | 24.22145 | 13.89 | 13.66 | 27.56 | 0.08 | 0.01 | 13.8 | 0 | 0.15 | 0 | 13.51 | 0 | 52.75591 | 45.89041 | 3 |
| 21 | 1006101 |  | 78 | 164.7 | 61 | m | 22.48758 | 5.07 | 10.15 | 15.22 | 1.23 | 1.25 | 2.6 | 0 | 0.86 | 0 | 9.28 | 0 | 58.7234 | 65.40284 | 2 |
| 22 | 1006201 |  | 55 | 168.4 | 64.7 | m | 22.81498 | 6.6 | 7.25 | 13.85 | 1.2 | 0.1 | 5.31 | 0 | 1.01 | 0.41 | 5.82 | 0 | 41.17647 | 36.2069 | 3 |
| 23 | 1006401 |  | 66 | 168 | 68 | m | 24.09297 | 11.63 | 10.01 | 21.64 | 0.16 | 0 | 11.47 | 0 | 0.93 | 0.01 | 9.06 | 0 | 46.90722 | 35.27132 | 3 |
| 24 | 1006501 |  | 77 | 164 | 61.8 | m | 22.97739 | 14.23 | 8.04 | 22.27 | 2.44 | 1.79 | 10.01 | 0 | 1.42 | 0.43 | 6.19 | 0 | 41.86047 | 42.85714 | 3 |
| 25 | 1006601 |  | 77 | 170.8 | 48.9 | m | 16.76228 | 5.53 | 5.22 | 10.74 | 1.78 | 0.68 | 3.07 | 0 | 1.41 | 0.36 | 3.44 | 0 | 55.21886 | 67.7686 | 2 |
| 26 | 1007101 |  | 82 | 164.1 | 70.3 | m | 26.10587 | 4.2 | 4.36 | 8.56 | 1.66 | 0.62 | 1.92 | 0 | 1.23 | 1.94 | 1.18 | 0 | 57.87037 | 63.45178 | 2 |
| 27 | 1007201 |  | 67 | 158.7 | 48.7 | m | 19.33638 | 30.58 | 24.66 | 55.24 | 8.27 | 2.16 | 20.14 | 0 | 6.07 | 2.25 | 16.34 | 0 | 32.59912 | 34.74178 | 3 |
| 28 | 1007301 |  | 62 | 168.4 | 78 | m | 27.50492 | 18.1 | 15.63 | 33.73 | 0.02 | 0 | 8.68 | 9.4 | 0.05 | 0 | 6.27 | 9.31 | 55.58442 | 78.96679 | 2 |
| 29 | 1007501 |  | 72 | 181.7 | 87 | m | 26.35175 | 8.77 | 7.95 | 16.73 | 0.67 | 0.03 | 8.08 | 0 | 0.96 | 0.07 | 6.92 | 0 | 69.04762 | 104.5902 | 1 |
| 30 | 1007601 |  | 71 | 163.6 | 67.1 | m | 25.07009 | 9.66 | 15.33 | 24.99 | 0.87 | 0.04 | 8.75 | 0 | 1.6 | 0.51 | 13.21 | 0 | 57.22543 | 88 | 1 |
| 31 | 1007801 |  | 78 | 181.9 | 59.4 | m | 17.95234 | 6.21 | 8.63 | 14.85 | 0.96 | 0.12 | 5.13 | 0 | 0.9 | 0.04 | 7.69 | 0 | 62.2291 | 69.31034 | 2 |
| 32 | 1008001 |  | 67 | 169.6 | 64.5 | m | 22.42374 | 7.31 | 9.33 | 16.64 | 1.97 | 0.59 | 4.74 | 0 | 1.6 | 0.87 | 6.85 | 0 | 51.93798 | 76.42586 | 2 |
| 33 | 1008201 |  | 83 | 169.3 | 70.7 | m | 24.66638 | 0.94 | 0.84 | 1.78 | 0.63 | 0.32 | 0 | 0 | 0.56 | 0.27 | 0 | 0 | 69.70803 | 87.61468 | 1 |
| 34 | 1008301 |  | 77 | 158.4 | 59 | m | 23.51482 | 11.66 | 15.01 | 26.68 | 0.83 | 0 | 10.84 | 0 | 1.62 | 0.56 | 12.56 | 0 | 64.70588 | 71.35135 | 2 |
| 35 | 1008401 |  | 75 | 167.2 | 68.7 | m | 24.57447 | 14.13 | 10.61 | 24.74 | 0.84 | 0 | 13.3 | 0 | 0.86 | 0.01 | 9.73 | 0 | 47.56098 | 55.45024 | 2 |
| 36 | 1008501 |  | 85 | 174.3 | 58.4 | m | 19.22286 | 4.71 | 6.57 | 11.28 | 1.49 | 0.43 | 2.79 | 0 | 1.39 | 0.09 | 5.08 | 0 | 49.16667 | 50 | 2 |
| 37 | 1008701 |  | 55 | 175.2 | 97 | m | 31.6012 | 7.81 | 11.64 | 19.44 | 1.35 | 0.15 | 6.31 | 0 | 1.24 | 0.5 | 9.89 | 0 | 61.53846 | 62.3053 | 2 |
| 38 | 1008801 |  | 69 | 181 | 85 | m | 25.94548 | 11.29 | 6.63 | 17.93 | 1.04 | 0.1 | 10.16 | 0 | 1.04 | 0.05 | 5.54 | 0 | 62.63345 | 56.77419 | 2 |
| 39 | 1009001 |  | 61 | 159 | 55.2 | m | 21.83458 | 13.17 | 22.45 | 35.62 | 2.34 | 0.3 | 4.79 | 5.74 | 3.76 | 1.07 | 1.15 | 16.47 | 40.41667 | 43.49776 | 3 |
| 40 | 1009501 |  | 69 | 162.2 | 63.4 | m | 24.09836 | 15.47 | 21.31 | 36.78 | 2.39 | 0.52 | 12.56 | 0 | 4.29 | 3.03 | 13.99 | 0 | 59.27835 | 50.66079 | 2 |
| 41 | 1010601 |  | 82 | 158.4 | 54.8 | m | 21.84088 | 7.86 | 17.69 | 25.55 | 1.36 | 0.13 | 6.37 | 0 | 1.83 | 0.75 | 5.98 | 9.14 | 45.02165 | 60.81871 | 2 |
| 42 | 2000101 |  | 58 | 183 | 98 | m | 29.26334 | 0.2 | 0.95 | 1.15 | 0.15 | 0.06 | 0 | 0 | 0.33 | 0.62 | 0 | 0 | 45.59859 | 65.40404 | 2 |
| 43 | 2000401 |  | 62 | 169 | 56 | m | 19.60716 | 25.52 | 37.66 | 63.17 | 11.15 | 11.32 | 2.89 | 0 | 18.02 | 17.13 | 1.75 | 0 | 26.90763 | 23.02405 | 4 |
| 44 | 2001902 |  | 86 | 153 | 59 | m | 25.20398 | 3.89 | 9.82 | 13.71 | 1.52 | 1.75 | 0.63 | 0 | 3.08 | 3.04 | 3.69 | 0 | 52.88889 | 103.4783 | 1 |
| 45 | 2002101 |  | 71 | 167 | 87 | m | 31.19509 | 1.49 | 1.62 | 3.11 | 1.13 | 0.36 | 0 | 0 | 0.78 | 0.84 | 0 | 0 | 51.11111 | 63.88889 | 2 |
| 46 | 2002201 |  | 68 | 168 | 59 | m | 20.9042 | 27.78 | 24.99 | 52.77 | 9.33 | 10.4 | 8.05 | 0 | 8.38 | 7.78 | 8.82 | 0 | 27.74566 | 35.95506 | 3 |
| 47 | 2002401 |  | 85 | 173 | 65 | m | 21.71807 | 1.93 | 9.43 | 11.36 | 0.97 | 0.96 | 0 | 0 | 3.62 | 3.44 | 2.37 | 0 | 52.14106 | 82.47012 | 1 |
| 48 | 2002901 |  | 61 | 160 | 60 | m | 23.4375 | 0.46 | 3.19 | 3.65 | 0.31 | 0.15 | 0 | 0 | 0.87 | 0.64 | 1.68 | 0 | 43.07692 | 59.82906 | 2 |
| 49 | 2003502 |  | 62 | 174 | 84 | m | 27.74475 | 3.14 | 2.72 | 5.85 | 1.76 | 0.56 | 0.82 | 0 | 1.08 | 0.63 | 1.01 | 0 | 48.18182 | 65.23077 | 2 |
| 50 | 2003801 |  | 82 | 159 | 61 | m | 24.12879 | 3.4 | 6.62 | 10.03 | 1.18 | 0.44 | 1.79 | 0 | 2.09 | 0.88 | 3.65 | 0 | 28.125 | 43.37349 | 3 |
| 51 | 2004301 |  | 80 | 172 | 61 | m | 20.61925 | 4.7 | 3.05 | 7.75 | 1.68 | 0.83 | 2.2 | 0 | 0.91 | 1.35 | 0.79 | 0 | 39.8773 | 50.19305 | 2 |
| 52 | 2004501 |  | 65 | 164 | 52 | m | 19.33373 | 0.67 | 1.74 | 2.41 | 0.54 | 0.13 | 0 | 0 | 0.88 | 0.86 | 0 | 0 | 49.02507 | 70.68273 | 2 |
| 53 | 2004601 |  | 66 | 164 | 64 | m | 23.79536 | 0.38 | 0.49 | 0.86 | 0.23 | 0.14 | 0 | 0 | 0.32 | 0.17 | 0 | 0 | 57.03422 | 60.97561 | 2 |
| 54 | 2004701 |  | 77 | 162 | 50 | m | 19.05197 | 11.43 | 15.74 | 27.17 | 3.46 | 2.24 | 5.73 | 0 | 5.05 | 1.89 | 8.79 | 0 | 36.36364 | 35.8209 | 3 |
| 55 | 2004801 |  | 68 | 157 | 60 | m | 24.34176 | 0.83 | 5.56 | 6.39 | 0.69 | 0.14 | 0 | 0 | 1.6 | 0.49 | 3.47 | 0 | 46.06061 | 78.35052 | 2 |
| 56 | 2005201 |  | 79 | 160 | 53 | m | 20.70313 | 26.04 | 10.54 | 36.59 | 16.49 | 7.14 | 2.32 | 0 | 6.13 | 3.32 | 1.09 | 0 | 38.3871 | 65.38462 | 2 |
| 57 | 2005701 |  | 52 | 180 | 98 | m | 30.24691 | 0.2 | 0.37 | 0.57 | 0.16 | 0.04 | 0 | 0 | 0.34 | 0.03 | 0 | 0 | 68.15534 | 89.08629 | 1 |
| 58 | 2006001 |  | 55 | 174 | 59 | m | 19.48738 | 26.8 | 23.88 | 50.68 | 14.33 | 11.47 | 1 | 0 | 12.08 | 6.81 | 4.98 | 0 | 33.03835 | 32.46377 | 3 |
| 59 | 2006901 |  | 56 | 167 | 77 | m | 27.60945 | 0.36 | 0.93 | 1.29 | 0.31 | 0.05 | 0 | 0 | 0.62 | 0.31 | 0 | 0 | 60.60606 | 60.81081 | 2 |
| 60 | 2007201 |  | 64 | 169 | 76 | m | 26.60971 | 3.92 | 3.99 | 7.9 | 1.52 | 0.28 | 2.12 | 0 | 1.22 | 0.21 | 2.55 | 0 | 57.54717 | 85.61404 | 1 |
| 61 | 2007601 |  | 78 | 166 | 68 | m | 24.67702 | 3.11 | 4.74 | 7.86 | 1.28 | 1.83 | 0 | 0 | 1.34 | 1.28 | 2.12 | 0 | 52.36686 | 78.66667 | 2 |
| 62 | 2008001 |  | 64 | 179 | 83 | m | 25.90431 | 0.73 | 0.41 | 1.14 | 0.43 | 0.29 | 0 | 0 | 0.27 | 0.14 | 0 | 0 | 62.84987 | 70.17045 | 2 |
| 63 | 2008201 |  | 76 | 157 | 43 | m | 17.44493 | 0.4 | 1.5 | 1.9 | 0.4 | 0 | 0 | 0 | 0.91 | 0.59 | 0 | 0 | 54.8913 | 59.06433 | 2 |
| 64 | 2008301 |  | 74 | 169 | 74 | m | 25.90946 | 4.23 | 1.1 | 5.32 | 1.83 | 1.01 | 1.38 | 0 | 0.67 | 0.42 | 0 | 0 | 67.02703 | 96.875 | 1 |
| 65 | 2008601 |  | 64 | 157 | 60 | f | 24.34176 | 0.26 | 0.18 | 0.44 | 0.22 | 0.03 | 0 | 0 | 0.15 | 0.03 | 0 | 0 | 66.38655 | 72.47706 | 2 |
| 66 | 2008801 |  | 59 | 175 | 60 | m | 19.59184 | 15.65 | 14.82 | 30.47 | 7.28 | 3.81 | 4.56 | 0 | 7.26 | 4.2 | 3.36 | 0 | 65.38462 | 95 | 1 |
| 67 | 2009101 |  | 67 | 158 | 64 | m | 25.63692 | 18.73 | 4.66 | 23.39 | 7.88 | 2.92 | 7.93 | 0 | 2.33 | 0.98 | 1.34 | 0 | 45.2381 | 55.88235 | 2 |
| 68 | 2009401 |  | 65 | 168 | 73 | m | 25.86451 | 6.8 | 2.38 | 9.18 | 4.08 | 0.98 | 1.75 | 0 | 1.48 | 0.89 | 0 | 0 | 62.52677 | 105.7971 | 1 |
| 69 | 2001501 |  | 60 | 157 | 68 | m | 27.58733 | 0.33 | 0.32 | 0.65 | 0.3 | 0.03 | 0 | 0 | 0.19 | 0.13 | 0 | 0 | 71.39108 | 125.3456 | 0 |
| 70 | 2001601 |  | 55 | 165 | 76 | m | 27.91552 | 2.17 | 2.72 | 4.89 | 1.41 | 0.76 | 0 | 0 | 0.84 | 0.56 | 1.31 | 0 | 69.93671 | 77.54386 | 0 |
| 71 | 2002001 |  | 59 | 168 | 95 | m | 33.6593 | 0.75 | 0.46 | 1.2 | 0.61 | 0.14 | 0 | 0 | 0.26 | 0.19 | 0 | 0 | 74.21203 | 88.3959 | 0 |
| 72 | 2006801 |  | 70 | 175 | 52 | m | 16.97959 | 0.02 | 0.2 | 0.22 | 0.02 | 0 | 0 | 0 | 0.16 | 0.04 | 0 | 0 | 71.12299 | 86.36364 | 0 |
| 73 | 2008101 |  | 60 | 164 | 68 | m | 25.28257 | 0.33 | 0.16 | 0.5 | 0.32 | 0.01 | 0 | 0 | 0.14 | 0.02 | 0 | 0 | 75.10373 | 68.56061 | 0 |
| 74 | 2009201 |  | 53 | 167 | 72 | m | 25.81663 | 0.32 | 0.29 | 0.61 | 0.23 | 0.09 | 0 | 0 | 0.09 | 0.2 | 0 | 0 | 73.07692 | 106.25 | 0 |
| 75 | 2009901 |  | 66 | 163 | 62 | m | 23.33547 | 2.42 | 1.82 | 4.24 | 1.08 | 0.47 | 0.88 | 0 | 0.82 | 1 | 0 | 0 | 75.37688 | 125 | 0 |
| 76 | 1007901 |  | 57 | 165.9 | 71.7 | m | 26.05112 | 6.03 | 5.18 | 11.21 | 1.07 | 0.07 | 4.89 | 0 | 1.65 | 0.96 | 2.57 | 0 | 79.08309 | 101.0989 | 0 |
